# Transumbilical single-port appendectomy in children: a retrospective study

**DOI:** 10.1007/s00383-026-06448-w

**Published:** 2026-05-12

**Authors:** Luísa Gomes Berto, Carolina Lopez da Silva, Marina Vieira Durante, Tales Rubens de Nadai, Marcos Antônio Marton

**Affiliations:** Graduate Program department, University of São Paulo, Bauru Campus, São Paulo, Brazil

**Keywords:** Appendicitis, Appendectomy, Laparoscopy, Minimally invasive surgical procedures, Pediatrics

## Abstract

**Objective:**

Acute appendicitis is the leading cause of abdominal surgery. This study evaluated outcomes of transumbilical single-incision laparoscopic appendectomy (TSILA) compared with conventional laparoscopy and open surgery.

**Methods:**

A retrospective study of 550 pediatric patients (0–18 years) undergoing an appendectomy (2020–2023) at a tertiary center was conducted. Patients were stratified by technique and appendicitis grade (I–IV). Data were analyzed using Pearson’s Chi-squared and ANOVA tests (*p* < 0.05).

**Results:**

TSILA accounted for 34.5% of cases (*n* = 190) and showed shorter hospital stays (2.98 days) compared to conventional laparoscopy (4.23 days) and open surgery (5.58 days) (*p* < 0.001). Postoperative fever was lower with TSILA (7.9%) compared to conventional laparoscopy (12.7%) and open surgery (27.5%) (*p* < 0.0001). Wound infection rates were similar between TSILA and conventional laparoscopy, both lower than open surgery. Postoperative antibiotic use was lowest with TSILA (*p* < 0.0001). In Grade IV, TSILA showed shorter hospital stay, with no significant differences in complications or antibiotic use.

**Conclusions:**

TSILA is a safe option, associated with shorter hospital stays and reduced antibiotic use in Grades I–III. In advanced cases, benefits were limited to shorter hospitalization, with outcomes comparable to conventional techniques.

## Introduction

Acute appendicitis is one of the most prevalent surgical emergencies worldwide, with an estimated mortality rate of 7 to 8%, and it remains the most common cause of abdominal surgery [1].

First performed in 1880 by Robert Lawson Tait [2] and later described by Charles McBurney in 1894, open appendectomy (OA) was widely used as the standard treatment for appendicitis [3], as it allows the surgeon direct access to the organ. However, this technique has gradually been replaced with the laparoscopic approach, due to its advantages of reduced surgical trauma, less postoperative pain, and smaller scars.

The introduction of laparoscopy for appendectomy was described by Kurt Semm in 1983 [4], and the first laparoscopic appendectomy for acute appendicitis was performed by Schreiber in 1987 [5].

In this context, transumbilical single-incision laparoscopic appendectomy (TSILA) is a single-incision technique that exteriorizes the appendix through the umbilicus for dissection and appendectomy, using a combined intra- and extra-abdominal approach [6]. This technique aims to reduce the size of the surgical incision, considering that in conventional laparoscopic surgery, the total length of the three trocar incisions is equivalent to that of an open procedure, while also offering additional advantages of minimally invasive surgery, such as decreased morbidity [7].

The procedure was first performed in adults by Pelosi and later executed in children by Begin [6]. In pediatric patients, TSILA is technically more feasible, since the distance between the umbilicus and the cecum is shorter in children than in adults, and their abdominal wall is more flexible, which facilitates exteriorization of the appendix through the umbilicus [8]. This, in turn, results in lower costs, shorter operative times, and better cosmetic outcomes for the surgical scar in pediatric patients [6].

Although the results appear promising, there is still limited evidence in the literature supporting its use in the pediatric population and in specific groups, such as obese children and adolescents. The present study aims to analyze retrospective data from a tertiary hospital where the procedure is routinely performed, comparing the main outcomes between groups and with data available in the literature.

## Methods

### Study type

This is a comparative retrospective longitudinal study based on the review of electronic medical records. The analysis included patients aged 0 to 18 years old who underwent an appendectomy between 2020 and 2023 in a Brazilian tertiary hospital. The study is comprised of the medical records of patients aged 0 to 18 years old who underwent an appendectomy, regardless of the surgical technique used, between 2020 and 2023. Electronic records were identified using the procedural codes corresponding to “acute appendicitis” and “unspecified appendicitis. Patients whose medical records contained missing data were excluded.

### Description of the surgical technique (TSILA)

Initially, a supraumbilical incision is performed, through which two juxtaposed 5 mm trocars are inserted. A 0° laparoscope is introduced through onde trocar, while a grasping instrument (grasper) is inserted through the other. The appendix is then identified, grasped, and mobilized toward the umbilical scar, where the final stage of the procedure is performed outside the abdominal cavity.

### Antibiotic protocol

Some patients were admitted to our service already receiving antibiotics prescribed at external institutions; these data were recorded when available. All patients who underwent an appendectomy received antibiotic prophylaxis with cefazolin at a dose of 30 mg/kg, administered one hour prior to the surgical incision. Postoperative antibiotic therapy was individualized according to the severity of appendicitis and each patient’s clinical condition.

### Surgical team expertise

The service is staffed by a team of five pediatric surgeons, all with more than 20 years of experience. Surgical procedures were performed by these specialists, along with the participation of first- and second-year residents from the general surgery residency program.

### Data collection

Data were organized into spreadsheets according to the following variables:

- Demographic data: age, sex, weight.
- Perioperative data: surgical time, use of prophylactic antibiotics (administered from 1 h before incision up to 48 h after surgery), length of hospital stay, histopathological diagnosis, conversion to conventional laparoscopy or open surgery.
- Postoperative complications: surgical site infection, postoperative fever, wound dehiscence, and use of therapeutic antibiotics (administered after 48 h post-surgery).

### Risks and benefits

The project was submitted to “Plataforma Brasil” and approved by the Research Ethics Committee of the Bauru School of Dentistry (FOB-USP) under formal opinion number 80121824.5.0000.5417. Patient consent was not required as the collected data were anonymous, thereby safeguarding the patient’s identity.

### Statistical analysis

The statistical analysis was conducted using a structured database comprising demographic variables (age, sex, weight), perioperative variables (operative time, use of antibiotic prophylaxis, length of hospital stay, histopathological diagnosis, need for procedural conversion), and postoperative outcomes (surgical site infection, postoperative fever, wound dehiscence, and requirement for therapeutic antibiotic therapy).

Data were initially organized in Microsoft Excel spreadsheets and subjected to descriptive statistical analysis, with means and standard deviations reported for normally distributed continuous variables, medians and interquartile ranges for non-normally distributed variables, and absolute and relative frequencies for categorical variables. The normality of continuous variables was assessed using the Shapiro–Wilk test. Group comparisons were performed using Student’s t-test for normally distributed continuous variables and the Mann–Whitney U test for non-parametric distributions. Categorical variables were compared using Pearson’s chi-square test or Fisher’s exact test, as appropriate based on expected frequencies.

All inferential analyses were carried out using IBM SPSS Statistics software, with technical support from the Statistics Center of the School of Medicine of Bauru (University of São Paulo). Additionally, considering a significance level of 5%, statistical power of 80%, and an estimated standard deviation of 29.6, a minimum sample size of 77 participants per group was calculated to ensure adequate power for the proposed comparisons.

## Results

### General analysis

A total of 550 medical records were analyzed. Table 1 presents the initial demographic characterization of the study population, employing descriptive statistical procedures to summarize the principal baseline variables. Categorical variables were reported as absolute frequencies, indicating that 307 patients were male (55.8%) and 243 were female (44.2%), demonstrating a slight predominance of males in the sample. Continuous variables were described using measures of central tendency, with a mean age of 9.65 years, suggesting that the cohort consisted predominantly of school-aged children and adolescents. The mean weight was 44.02 kg, consistent with the age distribution observed. These descriptive parameters serve to outline the baseline profile of the population and provide a reference framework for subsequent inferential analyses (Tables 2, 3).

**Table 1:** Summary table: demographic, clinical, surgical and postoperative characteristics

Variable	Category	*n*	%
Sex	Male	307	
Sex	Female	243	
Previous antibiotic use	Yes	38	6.9
Appendicitis grade	Grade I	198	36
Appendicitis grade	Grade II	144	26.2
Appendicitis grade	Grade III	63	11.5
Appendicitis grade	Grade IV	145	26.3
Surgical technique	Conventional laparoscopy	229	41.6
Surgical technique	TSILA	190	34.5
Surgical technique	Laparotomy	131	23.8
Wound dehiscence	Yes	20	3.6
Wound infection	Yes	43	7.8
Fever	Yes	80	14.6

**Table 2:** Technique × degree of appendicitis

Technique	Appendicitis grade	*n*	%
Conventional laparoscopy	Grade I	97	17.6
	Grade II	65	11.8
	Grade III	27	4.7
	Grade IV	40	7.3
Laparotomy	Grade I	21	3.8
	Grade II	22	4.0
	Grade III	13	2.4
	Grade IV	75	13.6
TSILA	Grade I	80	14.5
	Grade II	57	10.4
	Grade III	23	4.4
	Grade IV	30	5.5

Upon analyzing the information prior to hospitalization, it was found that the average time of history before admission was 53.45 h. Among the patients, 315 did not use antibiotics beforehand, 197 records did not contain information about the use of antibiotics before hospitalization, and 38 patients had used antibiotics previously.

Among the patients who had used antibiotics previously, 9 used amoxicillin alone, 1 used amoxicillin with clavulanate, and 1 used amoxicillin combined with azithromycin; 7 patients were treated with ceftriaxone, and 1 with the combination of ceftriaxone and metronidazole; 5 used metronidazole with amikacin and 1 used amikacin alone; 1 patient received cefepime; 3 used ciprofloxacin; 1 used nitrofurantoin; 1 used azithromycin alone; 1 used sulfamethoxazole-trimethoprim; 1 received benzathine penicillin; 1 used ketoprofen; and in 4 cases, there was no information recorded in the medical record.

The descriptive analysis shows that Grade I appendicitis was the most frequent presentation, accounting for 198 cases (36%), indicating a substantial proportion of early and uncomplicated inflammatory processes. Grade II appendicitis was observed in 144 patients (26.18%), whereas Grade IV appendicitis—associated with perforation or advanced inflammation—had a similar frequency, totaling 145 cases (26.36%). Grade III appendicitis was the least prevalent form, identified in 63 cases (11.46%). Overall, most patients (64%) presented with more advanced forms of the disease (Grades II–IV), while 36% had early-stage appendicitis.

The surgical techniques used in the appendectomies from the 550 medical records were classified as open laparotomy, TSILA, and conventional laparoscopy. Among the patients, 131 underwent open laparotomy, 190 underwent TSILA, and 229 underwent conventional laparoscopy.

The rate of wound dehiscence was low, occurring in 20 patients (3.6%), whereas the vast majority (96.4%) did not exhibit this outcome, suggesting adequate wound healing in most cases. Surgical site infection was identified in 43 patients (7.8%), while 92.2% of the sample remained free of infection, reflecting a relatively low incidence. Postoperative fever was documented in 80 patients (14.55%), whereas 470 patients (85.45%) did not present fever during the analyzed period. Overall, the data indicate low rates of early postoperative complications, with a predominance of favorable clinical outcomes.

The surgical technique used can be correlated with the degree of appendicitis (Table 2). Thus, in 97 cases of grade I appendicitis, 65 of grade II, 26 of grade III, and 40 of grade IV, surgeries were performed using the conventional laparoscopic technique. The open laparotomy technique was used in 21 cases of grade I appendicitis, 22 of grade II, 13 of grade III, and 75 of grade IV. Finally, the TSILA technique was used in 80 surgeries for grade I appendicitis, 57 with grade II, 24 with grade III, and 30 with grade IV.

### Statistical analysis

The statistical analysis revealed a statistically significant association between the surgical technique used and the occurrence of complications in the immediate postoperative period. Considering that the degree of appendicitis reflects the severity of inflammation and can influence both the choice of approach and clinical outcomes, the present study also evaluated the existence of an association between the surgical technique used, the degree of appendicitis, and the occurrence of complications in the immediate postoperative period.

In addition, the possible relationship between age, degree of appendicitis, and the incidence of complications was investigated, aiming to understand the impact of these clinical factors on surgical outcomes. The relationship between the length of hospital stay and surgical time was also analyzed concerning the technique used and the degree of appendicitis.

For the inferential analyses, Pearson’s Chi-squared Test (complication vs. technique) and ANOVA (length of hospital stay and operative time vs. technique) were used with a 5% significance level.

#### Correlation between postoperative complications, surgical technique, and degree of appendicitis

Postoperative fever was most prevalent in patients who underwent open laparotomy (27.5%), followed by conventional laparoscopy (12.7%) and TSILA (7.9%). The initial analysis using the Pearson’s Chi-squared test showed a statistically significant association between the surgical technique and the occurrence of fever (χ²=25,05; df = 2; *p* < 0,0001), suggesting an association between technique and this outcome.

However, recognizing the severity of appendicitis as a potential confounding factor, we performed a stratified analysis by grade (I–IV). The results showed a lack of statistical significance within each stratum: Grade I (*p* = 0.8015), Grade II (*p* = 0.9526), Grade III (*p* = 0.3078), and Grade IV (*p* = 0.3727). These findings suggest that the differences observed in the general analysis may be influenced by the unequal distribution of cases, as the open laparotomy group concentrated a higher proportion of Grade IV appendicitis, is associated with a greater risk of postoperative fever.

Similarly, surgical wound dehiscence presented rates of 7.6% with open laparotomy, 3.9% with conventional laparoscopy, and 0.5% with TSILA, with a statistically significant association in the general analysis (*p* = 0.0036). However, stratification by grade revealed no significant difference between techniques within each grade: Grade I (*p* = 0.1516), Grade II (*p* = 0.1554), Grade III (*p* = 0.5079), and Grade IV (*p* = 0.3960). The higher overall rate with open laparotomy reflects the concentration of patients with Grade IV appendicitis, who are more likely to present to dehiscence.

Surgical site infection also showed apparent differences between the techniques: 14.5% with open laparotomy, 5.7% with conventional laparoscopy, and 5.8% with TSILA, with a significant association in the general analysis (*p* = 0.0048). However, the stratified analysis by grade of appendicitis did not show a significant difference within each stratum: Grade I (*p* = 0.5723), Grade II (*p* = 0.7546), Grade III (*p* = 0.8515), and Grade IV (*p* = 0.1221). This observation suggests that the higher infection rate with open laparotomy was influenced by the higher proportion of severe cases treated in this group, and not by the technique itself.

The need for antibiotic therapy after 48 h post-procedure was significantly higher in patients who underwent open laparotomy (71%) and conventional laparoscopy (45.4%) compared to TSILA (25.3%), with a highly significant association in the general analysis (*p* < 0.0001). To control for the effect of the degree of appendicitis, the data were stratified. In grades I, II, and III, the surgical technique maintained a significant association with the need for antibiotics: Grade I (*p* = 0.0002), Grade II (*p* = 0.0099), and Grade III (*p* = 0.0029), with TSILA consistently associated with the lowest rates and open laparotomy with the highest. In cases of Grade IV appendicitis, there was no significant difference between techniques (*p* = 0.2025), indicating that in the most severe cases, the disease’s severity is the main determinant of the need for antibiotic therapy. In conclusion, the analysis demonstrates that the choice of surgical technique significantly influences the need for antibiotic therapy in less severe appendicitis, highlighting TSILA as the technique associated with the lowest rates.

**Table 3:** Statistical analysis

Appendicitis grade	Technique	Fever	Wound dehiscence	Wound infection	Therapeutic antibiotics
I	Laparotomy	1	1	0	9
	Conventional laparoscopy	4	1	2	20
	TSILA	2	0	3	5
	p-value	0.8015	0.1516	0.5723	0.0002
II	Laparotomy	2	0	1	10
	Conventional laparoscopy	5	3	6	26
	TSILA	4	0	4	10
	p-value	0.9526	0.1554	0.7546	0.0099
III	Laparotomy	2	0	1	11
	Conventional laparoscopy	5	1	1	20
	TSILA	1	0	1	8
	p-value	0.3078	0.5079	0.8515	0.0029
IV	Laparotomy	31	9	17	63
	Conventional laparoscopy	15	4	4	38
	TSILA	8	1	3	25
	p-value	0.3727	0.3960	0.1221	0.2025

#### Correlation between the length of hospital stay, degree of appendicitis, and surgical technique

The average length of hospital stay varied significantly according to the surgical technique adopted (*p* < 0.001), being longer for open laparotomy (5.58 days), followed by conventional laparoscopy (4.23 days), and TSILA (2.98 days). The analysis performed by two-way ANOVA showed that both the surgical technique and the degree of appendicitis significantly influence the length of hospital stay, as does the interaction between them (*p* < 0.001 for all), indicating that the effect of a technique is not uniform across all grades of appendicitis.

The Tukey HSD post-hoc analysis revealed that for less complicated appendicitis (Grades I, II, and III), there were no statistically significant differences between the techniques, suggesting that in these cases, the choice of technique does not substantially impact the duration of hospitalization. However, in the most severe cases (Grade IV), TSILA was associated with the shortest hospital stay, while conventional laparoscopy had an intermediate duration, and open laparotomy had the longest, demonstrating that for perforated appendicitis, suggesting that the choice of technique may influence hospital stay.

It is important to emphasize that, in this study, hospital discharge was determined and authorized by the same surgeon responsible for the operation, who also followed the patient throughout the entire hospitalization period. This approach minimized potential variability related to differences in management among medical teams, thereby ensuring greater homogeneity and reliability of the results. Discharge criteria included clinical improvement, absence of fever for at least 48 h, and adequate tolerance of oral intake.

In summary, hospital stay was influenced by both appendicitis severity and surgical technique, with TSILA associated with shorter hospital stay in Grade IV, while complication rates remained comparable between techniques.

#### Correlation between surgical time, degree of appendicitis, and surgical technique

The surgical time varied according to the technique used, with medians of 65 min for open laparotomy, 60 min for conventional laparoscopy, and 45 min for TSILA. Despite this numerical difference, ANOVA did not find a statistically significant association between the techniques (*p* = 0.308). In contrast, the degree of appendicitis was a determining factor in surgical time (*p* = 0.024), regardless of the technique used. The Tukey post-hoc analysis confirmed that the most severe cases were associated with longer surgical times: Grades II, III, and IV showed significantly longer times than Grade I, and Grade IV was significantly longer than Grade II. There was no significant interaction between technique and degree of appendicitis (*p* = 0.438), indicating that the prolongation of surgical time is primarily due to the severity of the disease and not the chosen surgical technique.

## Discussion

The results of this study support the safety and effectiveness of Transumbilical Single-port Laparoscopic Appendectomy (TSILA) in the treatment of pediatric acute appendicitis, showing favorable performance compared to conventional laparotomic and laparoscopic techniques. These findings are consistent with prior reports that highlight the advantages of minimally invasive approaches in pediatrics, including lower morbidity and faster recovery [1, 9].

Among the key outcomes evaluated, notable reductions were observed in hospital stay and antibiotic use, particularly in less severe cases (Grades I–III). In Grade IV appendicitis, TSILA was associated with shorter hospital stay, without significant differences in complication rates or antibiotic use. This finding reinforces the role of disease severity as a prognostic determinant, as previously described [1, 10], and suggests that the choice of surgical technique may influence clinical outcomes.

The analysis also demonstrated lower rates of immediate postoperative fever and surgical wound dehiscence with TSILA, which may reflect reduced surgical invasiveness associated with the single umbilical access. However, the wound infection rate did not differ from conventional laparoscopy, a result that confirms the technique’s safety but does not establish its absolute superiority over other minimally invasive modalities, consistent with findings by Esposito et al. [11] and Dorman et al. [12].

Another relevant aspect was the standardization of care, as all patients were operated on and followed up by the same surgical team, which was also responsible for authorizing hospital discharge. This methodological uniformity strengthens the validity of the findings by reducing potential biases related to divergent postoperative practices, as highlighted by Gorter et al. [13].

Despite the robustness of the sample and study design, some limitations must be acknowledged. This is a single-center study, which may restrict the generalizability of the results to other hospital contexts. Furthermore, TSILA is a technically more complex procedure when compared to the laparotomy technique and is dependent on a learning curve, which may limit its applicability in services with less experience in minimally invasive pediatric surgery.

## Conclusion

This study demonstrates that Transumbilical Single-port Laparoscopic Appendectomy (TSILA) is a safe and effective approach for managing acute appendicitis in children, with clinical outcomes varying by disease severity. TSILA was associated with shorter hospital stays and reduced antibiotic use, particularly in less severe cases (Grades I–III). In advanced cases (Grade IV), the primary benefit was limited to shorter hospitalization, with complication rates and antibiotic use comparable to conventional techniques. While these findings support the integration of TSILA into pediatric surgical practice, the limitations of this single-center study and the procedure’s technical complexity underscore the need for multicenter randomized trials to confirm these results.

## Data Availability

The data will be available upon request.
